# Pseudodiphallia: a rare kind of diphallia: A case report and literature review

**DOI:** 10.1097/MD.0000000000021638

**Published:** 2020-08-14

**Authors:** Wenchao Zhang, Nanze Yu, Zhifei Liu, Xiaojun Wang

**Affiliations:** Plastic and Reconstructive Surgery, Department of Plastic Surgery, Peking Union Medical College Hospital, China.

**Keywords:** diphallia, extrarenal pelvis, glans duplication, penis duplication, Pseudodiphallia

## Abstract

**Rationale:**

Pseudodiphallia, as a rare kind of diphallia, which is characterized by a small accessory penile-like tissue without a normal penile anatomy structure. Only a few cases have been reported. Here, we report a case of pseudodiphallia with phimosis and bilateral extrarenal pelvis.

**Patient concerns:**

A 23-year-old male visited our hospital with a complaint of external genitalia malformation. Physical examination revealed a normal-sized penis with phimosis, and an extra half glans horizontally attached to the right side of the normal glans penis is visible after completely retracting the foreskin. The CT urography showed a bilateral extrarenal pelvis, and no other abnormalities were found in the kidneys, ureter, bladder, and vertebral bodies.

**Diagnosis:**

Based on the physical examination and the CT urography results, the 23-year-old male was diagnosed with Pseudodiphallia.

**Intervention:**

Excessive penile tissue was resected, and a foreskin circumcision operation was performed under general anesthesia.

**Outcomes:**

The patient recovered smoothly without complications (no infection, hematoma, or wound dehiscence) after surgery. At 6 months follow-up, the patient was content with the external genitalia's appearance, and the urination and erectile function were normal.

**Lessons:**

Pseudodiphallia is a rare kind of diphallia, and this is the first report on pseudodiphallia with a bilateral extrarenal pelvis. CT urography can be used to assess the associated internal anomalies before surgery.

## 1 Introduction

Diphallia (penile duplication) is a rare congenital malformation with an incidence of about 1 per 5 to 6 million newborns.^[1,2]^ The severity of diphallia varies from a small accessory penile-like tissue to complete true penile duplication with other deformities, usually involving the urogenital, gastrointestinal, and musculoskeletal systems.^[3]^ Pseudodiphallia, as a rare kind of diphallia, characterized by a small accessory penile-like tissue without a normal penile anatomy structure. Patients with pseudodiphallia usually do not present with ureteral or renal malformations or other congenital deformities.^[4,5]^ Here, we report a case of pseudodiphallia with phimosis and bilateral extrarenal pelvis. Furthermore, we review previously published literature to further discuss the classification, etiology, and surgical methods of penile duplication.

## 2 Case report

**Figure 1:**
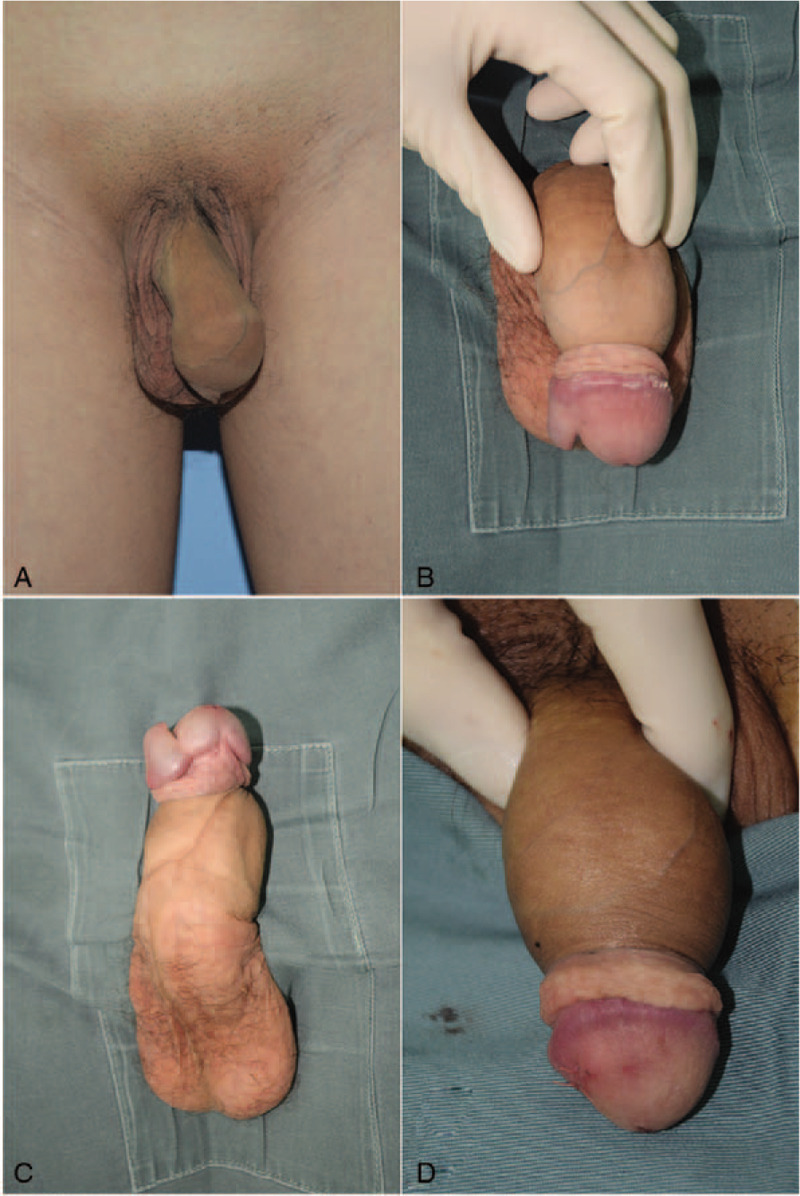
Physical examination revealed a normal-sized penis with phimois, an intact scrotum, 2 normally descended testicles, and a patent anus. After the fore skin was completely retracted, an extra half glan horizontally attached to the right side of the normal glans penis. There was only 1 urethral orifices which lies in the middle of the normal glans penis. And both of the normal penis and the extra half glan could be normal erected (A-C). Excessive penile tissue was resected and the foreskin circumcision operation was performed under general anesthesia (D).

A 23-year-old male visited our hospital with a complaint of external genitalia malformation. Physical examination revealed a normal-sized penis with phimosis, an intact scrotum, 2 normally descended testicles, and a patent anus. Upon completely retracting the foreskin, an extra half glans, horizontally attached to the right side of the normal glans penis, became visible. There was only 1 urethral orifice, which was located in the middle of the normal glans penis. Both the normal penis and the extra half glans could be normally erected (Fig. 1 A-C).

**Figure 2:**
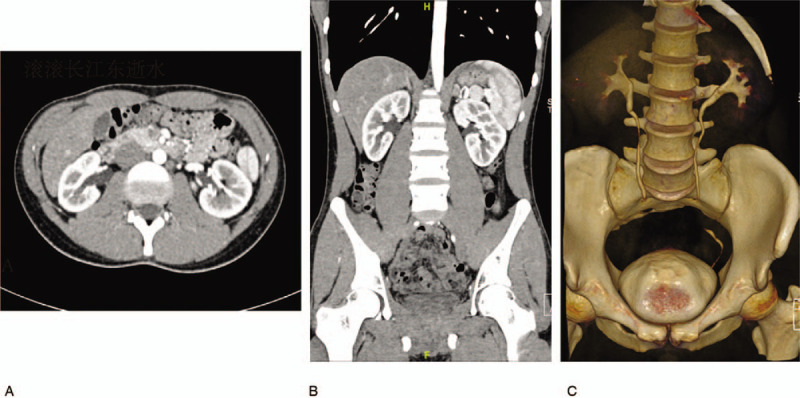
The CT urography showed bilateral extrarenal pelvis, and no other abnormalities were found in the kidneys, ureter, bladder, and vertebral bodies.

No apparent abnormalities were found in biochemical blood tests. The voiding cystourethrogram showed single urethras with a single bladder and no vesicoureteral reflux. The CT urography showed bilateral extrarenal pelvis, and no abnormalities were found in the kidneys, ureter, bladder, or vertebral bodies (Fig. 2).

**Figure 3:**
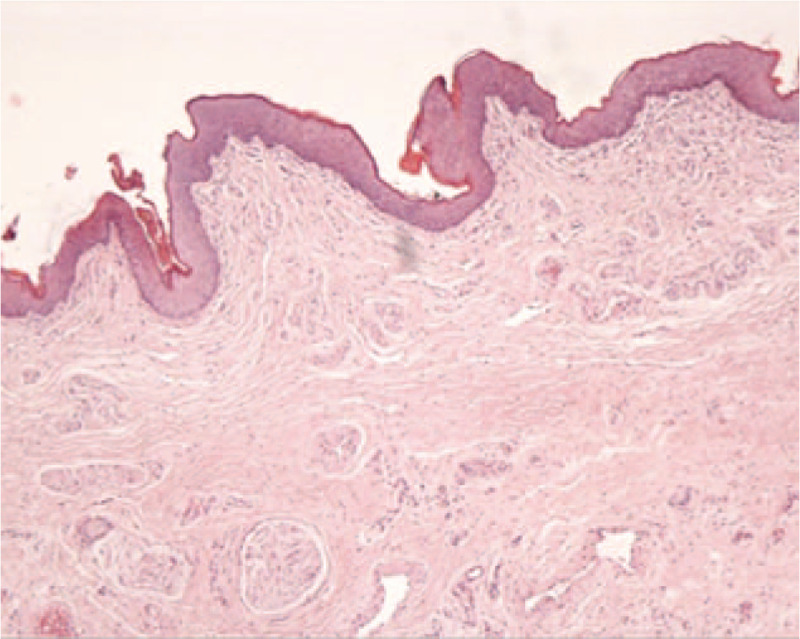
Tissue biopsy result revealed normal corpus cavernosum tissue.

Preoperatively, the patient was asked to clean the perineal region repeatedly and remove the smegma. We resected the excessive penile tissue and performed a foreskin circumcision operation under general anesthesia, after which the wound was sutured using a 5-0 absorbable suture (Fig. 1 D). No intraoperative or postoperative catheterization was performed. Oral estrogen was used to inhibit erection. A tissue biopsy revealed healthy corpus cavernosum tissue (Fig. 3). The patient recovered smoothly without complications (no infection, hematoma, or wound dehiscence) after surgery. At 6 months follow-up, the patient was content with the external genitalias appearance, and the urination and erectile function were normal.

Ethical approval was not necessary for this case report, and the patient provided informed consent for publication of the case.

## 3 Discussion

We searched for studies on diphallia published until February 2019 in various databases, including Pubmed, MEDLINE, and Embase. We excluded animal studies and papers without a full abstract. Since Wecker^[2]^ reported the first case in 1609, over 100 cases have been published. The summary of all literature found by using “pseudodiphallia” or “bifid phallus” as the keywords is shown in Table 1. Overall, after strict selection, we obtained 5 cases about true pseudodiphallia published until February 2019.

**Table 1:**
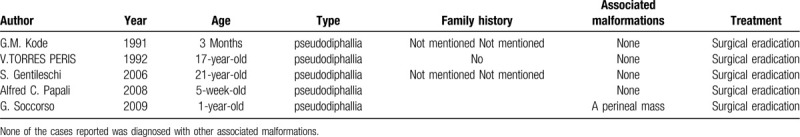
Clinical characteristics of pseudodiphallia in available reports.

### 3.1 Classification

The diphallia is a rare congenital malformation of the urinary system, varying from accessory penile-like tissue to complete penile duplication.^[6]^ Wecker reported the first case of diphallia in 1609; however, a wall painting from the Lupanar in Pompeii discovered in 1862, dating back to c. Seventy to 79 AD, depicts an entirely diphallic man, the god Priapus.^[7]^ In 1969, Schneider divided diphallia into 3 categories^[8]^:

1. diphallia of the glans alone,
2. bifid diphallia,
3. complete diphallia.

A later, more accepted, classification proposed by Aleem includes 2 main types^[2]^:

1. true diphallia
2. bifid phallus.

These 2 categories can be subdivided into 2 subclasses: complete and partial duplication.

In 2017, Jesus et al proposed a new simplified classification based on the clinical and surgical implications of each type^[5]^:

1. True penile duplication (each duplicate penis has 2 corpora and 1 spongiosum),
2. Hemiphalluses (each penis has corpora and a hemiglans),
3. Pseudoduplication (normal penis with an accessory penis-like tissue), and
4. Partial duplication (duplication involves only the distal penis).

By comparison, the term “True penile duplication” corresponds to “True diphallia”, “Hemiphalluses” corresponds to “Complete bifid phallus”, and “Partial duplication” corresponds to “Partial bifid phallus”. The term “pseudodiphallia” was initially proposed by Villanova and Raventos, referring to “true partial diphallia”.^[9]^ Recently, it was described as the malformation of a normal penis with accessory penis-like tissue. This is the least serious category, treated by resection of the extra tissue with no need for urogenital reconstruction.^[10]^ The latest classification is more accurate to understand the essence and the operative techniques for diphallia.

### 3.2 Clinical features

True duplications and hemiphalluses, especially true duplications, are always associated with other congenital defects, such as bladder and urethra duplication, kidney anomalies, exstrophy of the cloaca and bladder, imperforate anus, colon and rectosigmoid duplication, vertebral and limb anomalies, and ventral hernia.^[11–16]^ Thus, the mortality of infants increases due to a higher risk of genitourinary tract infections. In the case of pseudoduplications and partial duplications, there are usually no associated severe malformations. Patients with pseudoduplication only have extra penile tissue attached to the normal penis.^[5]^ The patient we treated was examined and was found to have a bilateral extrarenal pelvis, which has not been reported in the literature before.

### 3.3 Diagnostic testing

A comprehensive and detailed examination should be performed before treatment. A physical examination can aid in the detection of surface deformities, such as exstrophy of the cloaca and bladder, vertebral and limb anomalies, and imperforate anus. Ultrasonography, voiding cystourethrography, magnetic resonance imaging (MRI), and CT urography are used to assess the associated internal anomalies, such as bladder and urethra duplication, kidney anomalies, and colon and rectosigmoid duplication.^[17]^ MRI is a valuable method to accurately diagnose diphallia and associated malformations since T2-W images have a proper contrast resolution.^[18]^ In children, the erectile function can be tested by performing artificial erection with saline.^[5]^

### 3.4 Treatment

Treatment should be based on careful consideration of aesthetics, function, and ethics. In cases with other malformations, individualized surgery is usually performed step-by-step based on the specific defects. In general, associated malformations should be treated first.^[19]^ In the case of an actual penile duplication, partial duplication, and pseudoduplication, most surgeons choose to directly resect the hypoplastic duplicate penis, glans, or the accessory penile-like tissue in order to keep the main urethra. For hemiphalluses, more and more authors suggest joining the 2 penises without excision of excess tissue to preserve the excellent appearance and normal functionality of the external genitalia.^[20]^

### 3.5 Etiology

The etiology of diphallia remains unclear; however, there are many possible embryological explanations. In normal physiological conditions, bilateral cloacal tubercles join each other at the anterior end of the pars phallic. Afterward, mesodermal columns grow around the lateral margin of the cloacal plate from the genital tubercle.^[21]^ It is generally accepted that duplication of the penis is caused by a lack of fusion of the paired mesodermal anlagen of the genital tubercle by the 15th week of gestation.^[22]^ Almost all karyotype analyses of patients diagnosed with diphallia are found to be normal. However, 1 report discussed an infant whose blood chromosomal analysis revealed a balanced chromosome 46, XY, t(1;14)(p36.3;q24.3). The mothers blood chromosome analysis result was healthy, while his father was not available for a karyotype check.^[23]^ Although the first familial case of true diphallia was reported in 1994, diphallia is considered not familial or hereditary.^[24]^

Diphallia is a rare congenital malformation, varying from a small accessory penile-like tissue to complete penile duplication with other anomalies. The etiology of diphallia is unknown, and the treatment should be individualized based on careful considerations regarding the aesthetics, function, and ethics of the case. Pseudodiphallia is rare, but the least severe category of diphallia, which can be treated by resection of the extra tissue.

## Author contributions

**Conceptualization:** Nanze Yu.

**Data curation:** Wenchao Zhang, Nanze Yu, Xiaojun Wang.

**Formal analysis:** Nanze Yu.

**Investigation:** Wenchao Zhang, Zhifei Liu.

**Methodology:** Wenchao Zhang.

**Project administration:** Nanze Yu, Xiaojun Wang.

**Software:** Wenchao Zhang.

**Supervision:** Zhifei Liu, Xiaojun Wang.

**Visualization:** Zhifei Liu, Xiaojun Wang.

**Writing – original draft:** Wenchao Zhang, Nanze Yu, Zhifei Liu, Xiaojun Wang.

**Writing – review & editing:** Wenchao Zhang, Zhifei Liu.
